# The Human, Economic, Social, and Political Costs of COVID-19

**DOI:** 10.1007/978-981-33-6706-7_1

**Published:** 2021-03-03

**Authors:** Matteo Bonotti, Steven T. Zech

**Affiliations:** Politics and International Relations, Monash University, Melbourne, Australia

## Abstract

This chapter provides an overview of the human, economic, social, and political costs of COVID-19. The pandemic has had immediate negative health effects and is likely to also cause long-term health problems. In addition to economic repercussions across numerous sectors, COVID-19 has also had significant social and political effects. The chapter focuses on the strains that the pandemic has imposed on relationships between family members, friends, and romantic partners. It shows how COVID-19 has changed social practices in various everyday environments (e.g. restaurants, cafes, public transport), as the public has been forced to reimagine spaces and how to interact within them in ways that comply with new social distancing norms. The chapter also illustrates many of the political implications of COVID-19, including the way it has exacerbated ongoing political conflicts within and between states, compounded pre-existing international problems related to the movement of people, and affected levels of trust and political participation.

## Introduction

The origins and evolution of the novel coronavirus (COVID-19) can be traced back to the Chinese province of Hubei, where the first cases were identified in December 2019.^1^ The World Health Organization Director-General officially declared a global pandemic on 11 March 2020 as the virus spread rapidly around the world.^2^ The virus initially took hold in Western Europe and the United States, with disproportionate spread in specific high-traffic cities that serve as transportation hubs for international travel. Some studies suggest that SARS-CoV-2 genomes might have already been present in Spanish wastewater from as far back as March 2019^3^ and in Italy from December 2019.^4^ This chapter offers an overview of the human, economic, social, and political costs of the pandemic in order to prepare the ground for our analysis of the implications of COVID-19 for civility in the remainder of the book.

## The Human Cost

The human cost of COVID-19 is significant, yet its true scale is still uncertain. In addition to its immediate negative health effects, it is likely that the pandemic will also lead to a number of long-term health problems such as persistent pulmonary damage, post-viral fatigue, and chronic cardiac complications.^5^ Furthermore, researchers have already connected policies aimed at reducing the spread of COVID-19 through social isolation to other negative outcomes, such as a spike in suicide rates.^6^ Moreover, it has become apparent that COVID-19 is having disproportionate effects on specific subsections of the population in many of the countries affected. Factors may include age, race and ethnicity, class, and gender, among others. It is well known, for example, that older people face a higher risk of experiencing severe illness from COVID-19.^7^ In many countries, aged care facilities have become hotspots of infection and residents experienced higher-than-average death rates.^8^ Gender also seems to be a factor in mortality rates. There is growing evidence that men are more likely to die from COVID-19 than women, although the reasons are not yet clear.^9^ Furthermore, public health experts estimate disproportionate effects on maternal and child mortality rates in lower- and middle-income countries as a direct result of the virus, the subsequent strain on health systems, and reduced access to food.^10^

When it comes to race and ethnicity, some groups have been affected more than others. For example, black Americans have been disproportionately susceptible to infection and died at higher rates early on in the pandemic.^11^ The Center for Disease Control (CDC) in the US found that racial and ethnic minority groups have been particularly affected by COVID-19 due to such diverse factors as discrimination; low levels of health insurance, access, and service utilization; disproportionate representation in occupations with greater exposure to COVID-19; educational, income, and wealth inequalities; and housing conditions that render prevention strategies more difficult to implement.^12^ Other categories of vulnerable people who have been especially affected by COVID-19 include prisoners,^13^ as well as asylum seekers and refugees in camps and detention centres.^14^

Social class has also had a profound effect on the ability of people to protect themselves or recover from the virus. Data suggest, for example, that wealthier people have the resources to better adhere to social distancing policies and norms; are less likely to suffer from pre-existing health conditions; can more easily afford to stock up on food, medical, hygiene, and cleaning supplies; and are more likely to perform higher-skilled jobs that allow them to work from home.^15^ At the extreme end of the wealth spectrum, global elites have been able to stockpile supplies and make use of remote properties^16^ or yachts^17^ to isolate from broader society during the pandemic.

Socio-economic inequalities have had an impact on the effects of COVID-19 not only within individual countries, but also on a global scale. Many lower- and middle-income countries face significant economic contractions in terms of growth and income levels as a result of the pandemic.^18^ However, the challenges go beyond economic production and outputs. In some cases, healthcare systems already under stress have faced additional pressure. Furthermore, in the case of India, to cite just one example, the government has had a limited capacity to reach rural areas and experienced political pressure to limit testing to keep official case tallies low.^19^ The effects have been disastrous, with infection rates and death tolls well beyond the reported numbers. This will have carry-on effects on social welfare services aimed at those in need as resources are diverted to help combat the pandemic. But there are also success stories. Cuba, for example, has been able to respond to the pandemic promptly and efficiently, at least compared to other countries in the Caribbean and their Central and South American neighbours. Its free universal healthcare system proved crucial, combined with the highest doctor-to-population ratio in the world and the presence of an efficient national emergency planning structure.^20^

## The Economic Cost

In addition to the human costs, COVID-19 has also taken a significant toll on the global economy, particularly due to severe travel restrictions and lockdown measures aimed at reducing its spread. A significant number of workers across various sectors have lost their jobs, and this trend is likely to continue for the foreseeable future.^21^ From early on, the World Bank predicted the worst global recession since WWII, with the global economy expected to shrink drastically.^22^

The pandemic has also disrupted international trade relationships. For example, it rendered post-Brexit trade negotiations between the United Kingdom (UK) and the European Union (EU) more challenging^23^ and has exacerbated existing tensions in the trade relationship between the United States (US) and China.^24^ Furthermore, some regional markets (e.g. in Latin America) are experiencing significant economic downturns as a result of the pandemic.^25^ Moreover, financial markets have become increasingly volatile^26^ and the pandemic has also significantly disrupted global supply chains.^27^ Economic downturns in states like Victoria, Australia may be especially strong compared to other parts of the country. Waves of coronavirus cases have resulted in border closures that make interstate and international migration nearly impossible in Australia. The tourism, hospitality, and education sectors that rely heavily on migrant labour and international travel have been the most affected.^28^

Some sectors of the economy have been particularly affected by the pandemic and are experiencing significant contraction, as is the case with higher education. In some countries, such as the US, the UK, and Australia, many universities rely heavily (from a financial point of view) on the recruitment of international students, who generally pay higher enrolment fees than their domestic counterparts. Travel and visa restrictions during the pandemic have resulted in withdrawals and lower enrolment numbers among international students, with significant financial implications for the universities most affected.^29^

The creative arts sector has also struggled to cope with and adapt to the pandemic. Many music venues, theatres, and cinemas around the world were forced to keep their doors closed in the first half of 2020 due to social distancing rules and to reduce risks associated with the spread of COVID-19 in indoor environments. Some places reintroduced these measures during subsequent waves of the pandemic. The music industry has been significantly affected, with shows and festivals cancelled and album releases postponed.^30^ Unable to perform in person, production companies have had to reimagine theatrical performances for online audiences that still want to see a live show.^31^ Furthermore, many major theatres and opera houses across the world have made some of their performances freely available to the public via online streaming platforms.^32^ The global film industry has also been severely hit by the pandemic, with many cinemas closed, film festivals cancelled or moved online, and significant delays in the release of major motion pictures for fears that studios would be unable to recoup investments.^33^ The movie industry has been forced to evolve as movie theatre chains have responded to these challenges by negotiating agreements with movie studios on how to release films and charge audiences for access.^34^ In some cases, cinemas have tried to adapt to the new social distancing rules by rearranging their spaces and implementing strict health and safety checks.^35^ In other cases, we have witnessed unexpected changes, such as the revival of drive-in cinemas.^36^

The transportation sector will also feel the economic impact of the pandemic for the foreseeable future. Industry experts forecast a record-breaking financial loss for the commercial aviation sector. International flights in and out of many countries have been severely restricted, demand for air travel has plummeted, and airlines must take costly safety precautions to limit proximity to other passengers such as leaving middle seats empty. Cruise ship operators have not been immune to the pandemic either, especially due to people’s concerns regarding difficulties in abiding by social distancing rules in confined spaces.^37^ Likewise, those working in the ‘gig economy’ as drivers for rideshare services like Uber or Lyft face restrictions and lowered demand for service.^38^ Yet, some industry operators have benefitted from changing travel patterns and preferences among the general public. For example, sleeper trains have regained popularity among European travellers who are reluctant to fly between different cities and countries.^39^

Relatedly, the tourism industry faces unprecedented economic challenges due to travel restrictions and lower levels of disposable income among consumers who have been financially hit by the pandemic, resulting in hundreds of billions US$ in losses across the sector.^40^ The hotel industry has suffered similar hardships due to a sharp decline in hotel bookings.^41^ COVID-19 will force the entire tourism industry to rethink its focus and priorities to reduce susceptibility to shocks related to the pandemic and looming crises tied to global warming.^42^ The public may be forced to reimagine how it travels and start to prioritize local destinations, transforming the economic outlook of the sector.^43^

The restaurant and food services sectors have faced significant obstacles to profitability, and many businesses have been forced to shutter their doors. In some cases, the government has stepped in to force temporary closures or implement measures that require significant adjustments to a standard restaurant business model. Restaurants have had to contend with a severe reduction in consumer demand, a lower capacity to seat patrons, and unexpected expenditures to address safety concerns like adding plastic partitions to protect staff or redesigning seating arrangements, sometimes by prioritizing outdoors spaces.^44^ Many of them have adapted to this new environment by finding new ways to reach their customers. For example, an employee at one restaurant in Melbourne explained:

We knew that our restaurants would be very quiet so we immediately pushed our online orders when COVID-19 restrictions came into play. We’re lucky that we manufacture all our own pasta, sauces, pizzas and other products so pushing [these products] through clever marketing worked well for us. We introduced our ‘Door-to-Door Service’ which saw us visiting various suburbs on various days and this was very well received… It’s something our customers love and therefore something we’ll continue even when restrictions lift… We also decided to hold an online event. Like a dinner dance, but streamed online where customers purchase tickets to watch the entertainment and then they also have the option of purchasing a dinner pack that’s delivered to them before the event. This has also been well received.^45^

Following the easing of restrictions after the first wave of the pandemic, some governments stepped in to provide financial support for the industry by encouraging people to dine out.^46^ Cafes and the coffee industry have also been negatively impacted from an economic perspective.^47^ Conversely, grocery stores have generally benefited from changes in consumer behaviour as more people eat at home. However, they have also had to adapt their business model to the changing retail environment, prioritizing online shopping, expanding delivery services, and even exploring the potential to introduce mobile stores to replace brick-and-mortar markets.^48^

The sports industry has also been significantly affected by the pandemic, with major sporting events cancelled or postponed all over the world. Mass gatherings and large-scale events generate crowds where the risk of COVID-19 transmission rises exponentially. In March 2020, Japan’s Prime Minister and the president of the International Olympic Committee announced the postponement of the 2020 Tokyo Games, marking the first time the Olympics have been rescheduled for a reason other than war.^49^ The pandemic has forced diverse forms of professional and college sports leagues to halt play or devise alternative ways to reach audiences if they are to weather the storm. Many leagues and franchises have been unable to generate previous levels of advertising revenue because of postponements and find themselves in dire financial straits. Players themselves have been apprehensive about resuming play and moving forward with seasons. Sports like Major League Baseball could suffer losses in the billions of dollars, leading to tense negotiations between team owners and players regarding compensation and risk, with some asking whether athletes should be seen as ‘exploited workers or greedy millionaires’.^50^ US and Australian rules football leagues have faced similar challenges, with some players simply deciding to opt out.^51^ Football (soccer) leagues throughout Europe made the decision to simply suspend or cancel their seasons in 2020.^52^ When play resumed, it generally occurred behind closed doors. As sports teams and players suffer the financial costs of these disruptions, the public should be aware of the disparate capacity elite men’s clubs may have to contend with the financial challenges compared to women’s clubs, as is the case of English football.^53^

The agricultural sector has also faced considerable challenges. At the early stages of the pandemic, the prices of agricultural goods fell significantly, particularly due to lower demand from hotels and restaurants.^54^ While growing demand from grocery stores seems to have gradually offset those initial losses, farmers face new difficulties resulting from labour shortages and from the need to adapt to new social distancing rules.^55^ Labour shortages may also result in higher prices for fruit and vegetables.^56^ Furthermore, the meat industry has been particularly hard-hit by the pandemic. Many meat-processing plants have been the epicentres of COVID-19 outbreaks, resulting in shutdowns and meat shortages in the food supply chain.^57^

In addition to the areas examined in the foregoing analysis, other sectors that have been affected by COVID-19 include the manufacturing industry, the financial sector, the healthcare and pharmaceutical industry, and the real estate and housing sector.^58^ Other businesses on the economic fringes have also been hard-hit, especially those related to vice. For example, gambling hotspots in Las Vegas had to shutter their doors for some time, but gambling online has thrived even while sports betting has declined.^59^ Illicit drug trafficking and local distribution markets have faced novel challenges in supply chain and consumer demand too.^60^

Sex workers have also been affected by the social distancing restrictions implemented during the pandemic, given the central role that physical contact plays in the industry. Moreover, the stigma and discrimination that those working in this industry already experience has increased during the pandemic, further contributing to economic hardship across the sector. Some have responded by either demanding government support or by adapting their business model to emphasize other online services.^61^ The Australian Sex Workers Association clearly articulated the difficulties for sex workers who have been ‘placed in the impossible position of having to balance the need to protect [them]selves and the community against the prospect of no income and no access to financial relief’.^62^

## The Social Cost

In addition to its extensive economic implications, COVID-19 has also had a drastic effect on social life around the globe. Government measures related to social distancing rules, stay-at-home orders, business lockdowns, and curfews have in many cases eroded community relationships by drastically reducing opportunities for physical face-to-face interaction. These measures have significantly affected family life, both by increasing proximity among those forced to shared confined spaces during lockdowns^63^ and by keeping families apart to prevent risk of infection. For example, one grandmother we spoke with who lives in California described what interactions with her granddaughter looked like during the pandemic:

[My husband and I] were both looking at her and she’s looking at us and she’s hugging a dolly. And they’re through the glass. It was her birthday. And she came up to the glass, she puts her hand up [to ours] and she kissed the glass and I kissed the glass. We kissed each other through the glass and it was just heart-wrenching… I said, ‘I wish I could hug you, I miss you, I’m gonna throw you kisses’. We would go out into the yard and we stayed far away. We kind of did it all. [At first] we just did FaceTime. Then we started between the windows—we could at least see her there.^64^

Likewise, a grandmother in Italy whom we also interviewed explained:

The pandemic has taken away spontaneity from normal gestures of affection. There is fear but, at the same time, there is also a desire to hug grandchildren, children, and friends during the lockdown. [The pandemic] has taken away physical contact and people have had to replace this with video calls or messages, both with relatives and friends, in an attempt to exorcise fear.^65^

The pandemic has clearly rendered relationships among family and friends more difficult for many.^66^ However, it has also brought some people closer together thanks to greater flexibility in time schedules, alternative working arrangements, and reduced opportunities for other social activities.^67^ Relationships with both family and friends have also been sustained by the use of communication technologies during the pandemic.^68^ Furthermore, social media have played a key role in reducing isolation for both older^69^ and younger^70^ people, even though these platforms have also contributed to spreading rumours and misinformation.^71^ Romantic relationships and dating have also had to adapt to the new social distancing and travel restrictions. Some dating apps, for example, have altered user guidelines and introduced new video technology options so users can continue to interact with others while minimizing risks and adhering to social distancing guidelines.^72^ More generally, COVID-19 has had an impact on romantic love,^73^ and in some cases contributed to increasing stress among romantic partners, compounding factors that may lead to greater infidelity.^74^ Big social events like weddings had to be postponed in places like metropolitan Melbourne, Australia during its strict Stage 4 lockdown.^75^

Relationships between humans and non-human animals, and social practices surrounding them, have also been impacted. For example, data show that there has been a significant increase in pet ownership and adoption, as pets help reduce stress and loneliness, or encourage healthier and more active lifestyles.^76^ There has also been contention around the implications of the pandemic for certain animals. For example, the dog racing industry in Victoria, Australia saw an exemption from strict Stage 4 lockdown measures amid debates about potential animal welfare issues.^77^

The pandemic has also resulted in a housing crisis, as many people can no longer afford their rent or mortgage payments, thus risking eviction and homelessness.^78^ This has sometimes generated extreme and violent responses.^79^ In other cases, it has compounded pre-existing social harms like increased violence between intimate partners and other forms of abuse. Early indicators show that households in Brazil, Spain, the UK, and Cyprus saw spikes in domestic and family violence.^80^ A study in Dallas, Texas found a spike in domestic violence during the first two weeks of the stay-at-home order that subsided afterwards.^81^ The long-term isolation, stress, and uncertainty during the pandemic may also exacerbate alcohol and drug consumption. Furthermore, these conditions can increase the likelihood of relapse among recovering alcoholics and drug addicts too.^82^ There has also been a rise in online gambling.^83^

COVID-19 has also changed social practices in various everyday environments due to the need to re-imagine spaces and people’s interaction within them in ways that comply with social distancing norms.^84^ There are obvious logistical challenges to in-person education and how to manage students on school campuses. Options have included a combination of closures and social distancing practices.^85^ Educational institutions now rely on online learning to a greater degree, raising new challenges.^86^ For example, we spoke with a school teacher in Italy who explained that the transition to distance learning had several advantages, but suffered from a number of shortcomings. The new teaching format was not always suitable for younger pupils or students with disabilities. Furthermore, online teaching tended to sharpen the ‘digital divide’ between families with different levels of access to suitable spaces in the home, tablets, and highspeed Internet connections. He also described ongoing unruly behaviour and cheating among students, then added:

When our school reopened… the space was reorganized with single-seat desks… pupils always had to wear surgical masks and could only remove them in ‘static’ moments, sitting at their desks. They could not move nor could they pass materials among themselves… The interaction between teachers also profoundly changed. Teachers used to gather in the faculty lounge, which could no longer be used due to COVID-19. Opportunities for meetings and interactions with colleagues were clearly reduced; at the same time, teachers began to meet in online spaces like Google Meet, especially to share teaching practices. Yet, the ability to interact was decidedly reduced.^87^

Universities have also been forced to adjust courses and curricula for online delivery. While this is practically feasible, students may have fewer opportunities to participate in the off-line social networking that is crucial for career development.^88^ Furthermore, many universities may not survive the financial hit resulting from the pandemic.^89^

The pandemic has also affected the way people eat and drink. Restaurants, for example, have had to undergo several changes, including redesigning their spaces, accommodating lower numbers of customers in order to respect social distancing rules, making greater use of smart technologies (e.g. for menus and meal orders), and expanding their takeaway and delivery services.^90^ Some of them have adopted creative strategies in order to guarantee social distancing between patrons.^91^

 Likewise, government restrictions have forced some bars to close for extended periods of time in many locations. Those that have reopened or remained open had to reimagine how they serve customers and manage interactions between staff and patrons. Complex rules around indoor and outdoor spaces, as well as food service as it relates to the sale of alcohol, affect whether we visit these establishments and our experiences while there.^92^ An array of ‘multi-touch’ items like menus, salt and pepper shakers, cutlery, and coasters are now kept away from customers.^93^ One Irish pub in Spain’s Canary Islands used humour to communicate some of the real dangers associated with social practices in bars, putting up a notice that patrons should avoid singing the Neil Diamond hit ‘Sweet Caroline’ at all costs. Employees wrote some lyrics on a chalkboard explaining that, as a health precaution under COVID-19, ‘[t]here will be no: touching hands, reaching out, touching me, touching you’.^94^

Cafes have been forced to respond to the pandemic in creative ways as well, with some selling their stock as groceries and expanding their takeaway and delivery services.^95^ Furthermore, in many countries the pandemic has undermined the role of cafes as ‘third spaces’ between home and work, crucial for socializing and networking.^96^ The pandemic may have long-term effects on coffee culture around the globe.

Barbershops and hairdressers have also been at the epicentre of public debate concerning lockdown measures during the pandemic, with disagreement as to whether they constitute ‘essential’ businesses that should be exempt from lockdown restrictions. Barbershops traditionally serve important social functions for some cultural groups as spaces for community building, leisure and entertainment, gossip and local political engagement,^97^ as well as local education initiatives.^98^ They can also be important for men’s mental health.^99^ Likewise, hair salons can provide ‘a comforting source of self-care and community’^100^ and serve as ‘an important channel between members of the community and services such as family violence shelters’.^101^ This partly explains why many customers opposed and, in some cases, managed to revert government decisions to close down these businesses during the pandemic.^102^ In one extreme case, an armed militia group helped keep a barbershop open in a small US town in the state of Michigan.^103^

Beyond its direct effect on people’s health, COVID-19 has also indirectly affected people’s ability to stay healthy. For example, lockdown and social distancing restrictions aimed at reducing its spread have changed the way people exercise,^104^ with online streaming classes and programmes becoming a popular way for people to connect and participate in workout activities.^105^ Furthermore, when they have not been forced to close down, gyms have had to comply with strict health and safety measures, including the introduction of ‘hygiene marshals’.^106^ Partly due to risks associated with exercising in closed spaces, outdoor exercise has become increasingly popular.^107^ However, research shows that overeating and other unhealthy eating behaviours have also increased, thus posing additional challenges to individual and public health.^108^

The pandemic has affected other areas of social life related to leisure and recreation. Event-based social networks like Meetup, for example, have been forced to transition to virtual platforms in order to interact.^109^ A recent study in Australia found that activities within Meetup decreased by 86% during the pandemic. The researcher explains:

Participants in this study mentioned that Meetup was one of the main avenues in which they were exposed to new, potential relationships and that, due to lockdown measures, they had no way of expanding their social networks and thus making new friends. COVID-19 also had an amplifying effect on existing relationships within Meetup groups in the sense that close relationships became closer, and weak ones, weaker. Where relationships were strong enough, participants often used other social networking sites such as Facebook, WhatsApp and Instagram to maintain contact during lockdown, which highlights the importance of polymedia use.^110^

The way people travel for holidays and tourism has also changed.^111^ For example, both customers and business owners at beach destinations face unprecedented challenges that include new social distancing rules as well as stigma and public shaming for those who fail to respect them.^112^ People’s ability to access and experience national^113^ and local^114^ parks, as well as public spaces more generally,^115^ has also been deeply affected by the pandemic.

Travelling by public transport now includes additional demands to maintain social distance on crowded buses and subways. Passengers must also take new precautions to avoid handles and other surfaces that could spread the virus. Forward-thinking researchers will need to develop safer public transport infrastructure^116^ and new transport technologies to prevent an unsustainable shift back to a car-driven transport system.^117^ When it comes to pedestrians, proposed measures to contain the spread of the virus include touchless pedestrian crossings^118^ and crowd simulation technology to encourage social distancing.^119^ Rideshare services such as Uber have had to find ways of responding to reduced customer demand. For example, at times they have emphasized food delivery rather than taxi service to help keep drivers working and to mitigate issues of food insecurity.^120^ However, disruptions to their business model have had important social implications for sectors of the population with disabilities who normally depend on rideshare transportation services.^121^

The broader social effects of COVID-19 also concern the tensions that may arise between different individuals and social groups. Instances of social hoarding were particularly common at the onset of the pandemic, with people fighting over such products as toilet paper, hand sanitizer, flour, and pasta in shops and supermarkets.^122^ There have also been incidents of extreme rage over facemask policies, leading to the death of innocent bystanders and fatal confrontations with law enforcement.^123^ Furthermore, ageism and intergenerational tensions are on the rise in online spaces, especially between the ‘millennial’ and ‘baby boomer’ generations.^124^ Social stigma targeting infected people and those who have recovered from the illness, as well as doctors and health workers, has also become a widespread phenomenon.^125^ COVID-19 has also fuelled racism and xenophobia.^126^ Hate speech, hate crimes, and discriminatory practices targeting people with Chinese and East Asian backgrounds,^127^ Muslims,^128^ Jews,^129^ and Romani communities^130^ have been especially common. At the international level, the pandemic has generated negative attitudes towards countries with high levels of infections.^131^ One study, for example, revealed spikes in incivility directed at China on South Korean social media.^132^

## The Political Cost

The global pandemic has generated a range of international and domestic political problems. The COVID-19 health crisis constitutes an exogenous shock to the broader international system, disrupting international politics and creating new tensions between adversaries and allies alike. It will undoubtedly have profound implications for and lasting effects on geopolitics for years to come.^133^ Political leaders from major powers like the US and China may seek to use the crisis to find advantage in an ongoing contest for hegemony in the global political order.^134^ In many contexts, states have been left scrambling to secure sufficient supplies and resources to effectively contend with the virus, prioritizing national interest and the well-being of their own citizens. The US, for example, requested that the firm 3M refrain from selling protective masks to Canada and countries in Latin America to keep them for domestic use.^135^ A form of ‘vaccine nationalism’ took hold in a race to develop a vaccine for the virus that created barriers to cooperation and prioritized domestic delivery when mass production got underway.^136^

The pandemic has the potential to exacerbate ongoing political conflicts between states. For example, COVID-19 risks inflaming tensions between India and Pakistan over Kashmir. As political leaders in both countries focus on fighting the virus, we could see further entrenchment of the militarized status quo, as well as local efforts to highlight the inadequacy of Indian governance in Kashmir. There is the potential that hardline Indian nationalist policies might be used to divert public attention from the COVID-19 crisis. However, the scale of the pandemic threat will most likely shift attention in India and Pakistan to the immediate demands of public health services and the need to alleviate economic hardship domestically.^137^

Polities with supranational governance structures like the European Union have experienced discord over new policies. EU member states eventually managed to compromise on an economic recovery plan in July 2020, despite tensions during the negotiation process, especially due to concerns of so-called ‘frugal’ countries about the cost of the plan.^138^ However, tensions within the EU have also been driven by disputes concerning seasonal migrant labour, with some business, especially farmers, demanding access to foreign workers, and some populist leaders calling instead for tighter restrictions on immigration.^139^

The pandemic has also compounded pre-existing international problems related to the movement of people. Asylum seekers and refugees have been particularly affected,^140^ especially since the pandemic risks exacerbating existing humanitarian crises.^141^ The pandemic has also had an impact on temporary economic migrants, particularly as a result of the economic downturn that has forced many companies to lay off employees. Even when governments have introduced economic measures to support businesses, temporary migrants have often been excluded from these programmes.^142^ Some governments are also considering changes to migration rules^143^ and taking drastic steps in modifying the way they address asylum claims, including limitations to face-to-face interviews, introducing new physical barriers, or even encouraging applicants to ‘bring [their] own black or blue ink pens’.^144^ Internal migration has also been affected by the pandemic, as many governments have imposed restrictions on internal travel.^145^

The public health crisis is also affecting domestic political divisions in multiple contexts. For example, during post-Brexit negotiations between the UK and the EU, some politicians exploited the pandemic for partisan political gain.^146^ In some cases, politicians have challenged the authority of experts, undermining citizens’ trust in evidence-based knowledge.^147^ They have also mischaracterized or appropriated scientific expertise around issues like mask wearing to advance their positions.^148^ Debate about the pandemic in some countries has been driven by and exacerbated pre-existing political polarization, stoking tensions between regional/state and national/federal political authorities. However, calls for unity and coordinated action has sometimes also helped to reduce ideological and partisan divides.^149^

The pandemic poses unique challenges to state stability and could compound risks of political violence, internal armed conflict, and incidents of state failure. Rebel groups and other militant actors have seized opportunities to expand control, advance political objectives, and demonstrate a capacity to govern and enforce rules. For example, armed actors operating along the southwest coast of Colombia made public declarations that curfew violators would be treated as ‘military targets’.^150^ COVID-19 has provided a chance for armed opposition groups to scale up attacks and target government opponents in some cases, while in others groups have seized on the opportunity to improve claims of legitimacy and demonstrate their capacity to provide public services and govern. For example, the Islamic State, the Taliban, and al-Qaeda affiliates have all provided guidance and local support to contend with the pandemic.^151^

Political participation has also been affected by the pandemic. Protest politics, for example, has been at the epicentre of public debate. On the one hand, citizens in some countries have taken to the streets to protest against government restrictions to contain the virus, such as lockdown and stay-at-home orders.^152^ On the other hand, protests such as those organized by Black Lives Matter activists around the world became a topic of contention as citizens and political leaders disagreed as to whether those gatherings may have contributed to new COVID-19 outbreaks.^153^

The effects on political participation also extend to electoral politics. For example, in some countries local and national political authorities decided to postpone elections^154^ or reimagine electoral procedures and practices. Governments have taken steps like increasing the use of postal voting^155^ or introducing measures to guarantee social distancing, health, and safety during the voting process.^156^ There has also been an impact on campaign practices due to the need to restrict traditional rituals and habits like shaking hands.^157^ Furthermore, political rallies constitute extreme health risks for the spread of the virus.^158^ This point became especially prominent after former US President Donald Trump resumed large political campaign events shortly after his hospitalisation from COVID-19 treatment.^159^ Other politicians experimented with virtual rallies and events to mark important milestones in campaigns like the Democratic Party’s announcement of a presidential candidate in August 2020.^160^ The content of political campaigns and party politics has also evolved as a result of COVID-19. Issues such as public health and socio-economic and racial inequality, for example, have become more salient,^161^ and parties traditionally divided over fiscal responsibility and public spending have sometimes converged on more similar positions.^162^

Trust is an important aspect of political life as it relates to politicians, law enforcement, and the media, among others. High-profile incidents of politicians who ignore their own stay-at-home orders^163^ or who publicly contradict or undermine health experts^164^ can lead to general confusion and the erosion of trust in public officials. The politicization of issues like mandatory mask wearing illustrates how a lack of consensus and divergent policies can frustrate public health measures and lead to greater distrust not only towards politicians but also towards law enforcement officials tasked with ensuring compliance. In extreme cases, law violators have lashed out in violence against police officers enforcing these new laws.^165^ In a particularly sensational case, members of an extremist militia were arrested in relation to alleged plans to kidnap Michigan’s Governor and put her on ‘trial’ for restrictive pandemic policies.^166^ Furthermore, the media can have a compounding effect on public trust (or lack thereof), by employing framing techniques^167^ or prioritising specific content as they deliver information to the public.^168^ Social media can further complicate political trust, as they are a popular channel for politicians to spread misinformation about COVID-19 and related policies.^169^

## Conclusion

This chapter provided an overview of the human, economic, social, and political costs of the pandemic. The world faces unprecedented challenges related to COVID-19, including an immense strain on relationships and the way people interact with one another in different aspects of their lives. Uncertainty and disruptions to social and political life will require a better understanding as to how the broader public needs to prepare and respond. Politicians and other decision-makers will face increasing pressure to come up with policies that are effective at containing the pandemic, limiting its economic impact, and minimising harmful social and political consequences. They face the difficult task of balancing diverse interests, values, and demands, while also having to ensure that they rely on sound scientific evidence. In the remainder of this book, we will examine all these challenges through the lens of civility.

### Notes

1. Liangsheng Zhang et al., ‘Origin and Evolution of the 2019 Novel Coronavirus’, *Clinical Infectious Diseases*, Vol. 71, No. 15 (2020): 882–883. 10.1093/cid/ciaa112.
2. Jin Wu et al., ‘How the Virus Got Out The New York Times’, *The New York Times*, 22 March 2020. https://www.nytimes.com/interactive/2020/03/22/world/coronavirus-spread.html.
3. Gemma Chavarria-Miró et al., ‘Sentinel Surveillance of SARS-CoV-2 in Wastewater Anticipates the Occurrence of COVID-19 Cases’, *MedRxiv*, published online on 13 June 2020, 1–10. https://doi.org/10.1101/2020.06.13.20129627.
4. Giuseppina La Rosa et al., ‘SARS-CoV-2 Has Been Circulating in Northern Italy Since December 2019: Evidence from Environmental Monitoring’, *MedRxiv*, published online on 2020. https://doi.org/10.1101/2020.06.25.20140061.
5. Evelyn Lewin, ‘What Are the Long-Term Health Risks Following COVID-19?’, *NewsGP*, 24 June 2020. https://www1.racgp.org.au/newsgp/clinical/what-are-the-long-term-health-risks-post-covid-19.
6. Leo Sher, ‘The Impact of the COVID-19 Pandemic on Suicide Rates’, *QJM: An International Journal of Medicine*, Vol. 113, No. 10 (2020): 707–712. 10.1093/qjmed/hcaa202.
7. Anonymous, ‘Coronavirus Disease 2019 (COVID-19)’, *Centers for Disease Control and Prevention*, 11 February 2020. https://www.cdc.gov/coronavirus/2019-ncov/need-extra-precautions/older-adults.html.
8. Anonymous, ‘Victoria Records Its Second-Deadliest Day as 87 Aged Care Homes Battle Coronavirus Outbreaks’, *ABC News*, 29 July 2020. https://www.abc.net.au/news/2020-07-29/victoria-coronavirus-aged-care-deaths-rise-as-cases-up-295/12502298.
9. Clare Wenham, Julia Smith, and Rosemary Morgan, ‘COVID-19: The Gendered Impacts of the Outbreak’, *The Lancet*, Vol. 395, No. 10227 (2020): 846–848. 10.1016/S0140-6736(20)30526-2; John Ng et al., ‘COVID-19 Mortality Rates by Age and Gender: Why Is the Disease Killing More Men than Women?’, *RGA*, 10 July 2020. https://rgare.com/knowledge-center/media/research/covid-19-mortality-rates-by-age-and-gender-why-is-the-disease-killing-more-men-than-women.
10. Timothy Roberton et al., ‘Early Estimates of the Indirect Effects of the COVID-19 Pandemic on Maternal and Child Mortality in Low-Income and Middle-Income Countries: A Modelling Study’, *The Lancet Global Health*, Vol. 8, No. 7 (2020): e901–908. 10.1016/S2214-109X(20)30229-1.
11. Akilah Johnson and Talia Buford, ‘Early Data Shows African Americans Have Contracted and Died of Coronavirus at an Alarming Rate’, *ProPublica*, 3 April 2020. https://www.propublica.org/article/early-data-shows-african-americans-have-contracted-and-died-of-coronavirus-at-an-alarming-rate.
12. Ed Pilkington, ‘Black Americans Dying of Covid-19 at Three Times the Rate of White People’, *The Guardian*, 21 May 2020. http://www.theguardian.com/world/2020/may/20/black-americans-death-rate-covid-19-coronavirus; Erin K. Stokes, ‘Coronavirus Disease 2019 Case Surveillance—United States, January 22–May 30, 2020’, *MMWR. Morbidity and Mortality Weekly Report*, Vol. 69, No. 24 (2020): 759–765. 10.15585/mmwr.mm6924e2; Michelle Roberts, ‘Coronavirus: Risk of Death Is Higher for Ethnic Minorities’, *BBC News*, 2 June 2020. https://www.bbc.com/news/health-52889106; Anonymous, ‘Disparities in the Risk and Outcomes of COVID-19’, *Public Health England*, 2 June 2020, 19. https://www.gov.uk/government/publications/covid-19-review-of-disparities-in-risks-and-outcomes.
13. Nerita Waight, ‘Hundreds Have Died in US Prisons from Covid-19. Will Australia Act Before It’s Too Late?’, *The Guardian*, 30 July 2020. http://www.theguardian.com/commentisfree/2020/jul/30/thousands-have-died-in-us-prisons-from-covid-19-will-australia-act-before-its-too-late.
14. Harriet Spinks, ‘Seeking Asylum in the Time of Coronavirus: COVID-19 Pandemic Effects on Refugees and People Seeking Asylum’, *Parliament of Australia*, 19 May 2020. https://www.aph.gov.au/About_Parliament/Parliamentary_Departments/Parliamentary_Library/FlagPost/2020/May/COVID-19_-_impacts_on_refugees_and_asylum_seekers.
15. Richard V. Reeves and Jonathan Rothwell, ‘Class and COVID: How the Less Affluent Face Double Risks’, *Brookings*, 27 March 2020. https://www.brookings.edu/blog/up-front/2020/03/27/class-and-covid-how-the-less-affluent-face-double-risks/; Anonymous, ‘The Social Impact of COVID-19’, *United Nations*, 6 April 2020. https://www.un.org/development/desa/dspd/2020/04/social-impact-of-covid-19/.
16. Olivia Carville, ‘“We Needed to Go”: Rich Americans Activate Pandemic Escape Plans’, *Bloomberg*, 19 March 2020. https://www.bloomberg.com/news/articles/2020-04-19/-we-needed-to-go-rich-americans-activate-pandemic-escape-plans; Katie Canales, ‘The Wealthiest of Silicon Valley Have Become Super Doomsday Preppers by Buying Remote New Zealand Properties, Getting Eye Surgeries, and Stockpiling Ammo and Food’, *Business Insider Australia*, 14 March 2020. https://www.businessinsider.com.au/silicon-valley-doomsday-preppers-new-zealand-2020-3.
17. Sarakshi Rai, ‘Outrage as Billionaire David Geffen Posts Drone Shot of Luxury Yacht During His “Perfect Isolation”’, *Esquire Middle East*, 2 April 2020. https://www.esquireme.com/content/45078-outrage-as-billionaire-david-geffen-posts-drone-shot-of-luxury-yacht-during-his-perfect-isolation.
18. Anonymous, ‘The Global Economic Outlook During the COVID-19 Pandemic: A Changed World’, *World Bank*, 8 June 2020. https://www.worldbank.org/en/news/feature/2020/06/08/the-global-economic-outlook-during-the-covid-19-pandemic-a-changed-world.
19. Amy Kazmin, ‘Modi Stumbles: India’s Deepening Coronavirus Crisis’, *Australian Financial Review*, 28 July 2020. https://www.afr.com/world/asia/modi-stumbles-india-s-deepening-coronavirus-crisis-20200728-p55g42.
20. Emily Morris and Ilan Kelman, ‘Coronavirus Response: Why Cuba Is Such an Interesting Case’, *The Conversation*, 15 April 2020. http://theconversation.com/coronavirus-response-why-cuba-is-such-an-interesting-case-135749. It is also important to stress, however, that other factors such as housing shortages and poor infrastructure have partly limited these positive effects.
21. Anonymous, ‘Coronavirus Causes 150 Million Job Losses Largest Greenhouse Gas Emissions Drop in History’, *The Australian*, 10 July 2020. https://www.theaustralian.com.au/business/economics/coronavirus-causes-150-million-job-losses-largest-greenhouse-gas-emissions-drop-in-history/news-story/f9a35f56fb71db5475b801c995b73acc.
22. Mark Felsenthal, ‘COVID-19 to Plunge Global Economy into Worst Recession Since World War II’, *World Bank*, 8 June 2020. https://www.worldbank.org/en/news/press-release/2020/06/08/covid-19-to-plunge-global-economy-into-worst-recession-since-world-war-ii.
23. Rebecca Christie, ‘How Will COVID-19 Impact Brexit? The Collision of Two Giant Policy Imperatives’, *Bruegel*, 19 May 2020. https://www.bruegel.org/2020/05/how-will-covid-19-impact-brexit-the-collision-of-two-giant-policy-imperatives/.
24. Anonymous, ‘How Will COVID-19 Impact US-China Trade Relations?’, *Open Access Government*, 28 May 2020. https://www.openaccessgovernment.org/us-china-trade-relations/87533/.
25. Anonymous, ‘UN Policy Brief: The Impact of COVID-19 on Latin America and the Caribbean’, July 2020. https://www.un.org/sites/un2.un.org/files/sg_policy_brief_covid_lac.pdf.
26. Anonymous, ‘Allianz | COVID-19: Contagion Risks Also Apply to Markets’, *Allianz*, 15 July 2020. https://www.allianz.com/en/economic_research/publications/specials_fmo/15072020_CapitalMarkets.html.
27. David Laborde et al., ‘COVID-19 Risks to Global Food Security’, *Science*, Vol. 369, No. 6503 (2020): 500–502. 10.1126/science.abc4765.
28. Michael Janda, ‘Victorian Economy Hardest Hit by Coronavirus Fallout as Immigration Dries Up’, *ABC News*, 6 July 2020. https://www.abc.net.au/news/2020-07-06/victorian-economy-hardest-hit-by-coronavirus-fallout/12426234.
29. Paul Karp, ‘Australian Universities Facing $16bn Black Hole as Covid-19 Student Numbers Plummet’, *The Guardian*, 3 June 2020. http://www.theguardian.com/australia-news/2020/jun/03/australian-universities-facing-16bn-black-hole-as-covid-19-student-numbers-plummet; Anonymous, ‘The Impact of Coronavirus on Higher Education’, *Times Higher Education (THE)*, 15 April 2020. https://www.timeshighereducation.com/hub/keystone-academic-solutions/p/impact-coronavirus-higher-education.
30. Rolling Stone, ‘How Coronavirus Is Wreaking Havoc on Music’, *The Rolling Stone*, 27 April 2020. https://www.rollingstone.com/pro/lists/coronavirus-music-business-latest-974262/.
31. Kelsey Jacobson, ‘Theatre Companies Are Pushing Storytelling Boundaries with Online Audiences Amid COVID-19’, *The Conversation*, 22 July 2020. http://theconversation.com/theatre-companies-are-pushing-storytelling-boundaries-with-online-audiences-amid-covid-19-141583.
32. Anonymous, ‘Nightly Met Opera Streams’, *The Metropolitan Opera*, 2020. https://www.metopera.org/user-information/nightly-met-opera-streams/.
33. Steven Zeitchik, ‘Disney Shifts “Mulan” as Hollywood Throws in the Towel on July’, *The Washington Post*, 27 June 2020. https://www.washingtonpost.com/business/2020/06/26/disney-mulan-move-covid/; Helen W. Kennedy and Sarah Atkinson, ‘How the Movie Industry Is Fighting Lockdown’, *The Conversation*, 23 May 2020. http://theconversation.com/how-the-movie-industry-is-fighting-lockdown-139149.
34. Chris Lee, ‘What AMC and Universal’s Historic Streaming Deal Really Means for Moviegoers’, *Vulture*, 29 July 2020. https://www.vulture.com/2020/07/amc-and-universals-historic-streaming-deal-explained.html.
35. Anonymous, ‘How Movie Theaters Hope to Adapt for Social Distancing’, *NBC Nightly News*, 29 May 2020. https://www.youtube.com/watch?v=d6i5kQeNePY&ab_channel=NBCNews.
36. Sue Hewitt, ‘How COVID-19 Saved the Drive-in Cinema’, *RACV*, 16 June 2020. https://www.racv.com.au/royalauto/living/at-home/drive-in-cinema-comeback.html.
37. John Gradek, ‘How COVID-19 Could Impact Travel for Years to Come’, *The Conversation*, 4 August 2020. http://theconversation.com/how-covid-19-could-impact-travel-for-years-to-come-142971.
38. Veena Dubal and Meredith Whittaker, ‘Uber Drivers Are Being Forced to Choose Between Risking Covid-19 or Starvation’, *The Guardian*, 25 March 2020. http://www.theguardian.com/technology/2020/mar/25/uber-lyft-gig-economy-coronavirus; Jack Kelly, ‘Uber and Lyft Are on a Collision Course with the Effects Of COVID-19 and New California Lawsuit That Could Crush Their Business Model’, *Forbes*, 8 May 2020. https://www.forbes.com/sites/jackkelly/2020/05/08/uber-and-lyft-are-on-a-collision-course-with-the-effects-of-covid-19-and-new-california-lawsuit-that-could-crush-their-business-model/#3c61397e20d4.
39. Daniel Boffey, ‘“People Don’t Want to Fly”: Covid-19 Reawakens Europe’s Sleeper Trains’, *The Guardian*, 27 July 2020. https://www.theguardian.com/world/2020/jul/27/covid-19-reawakens-europe-sleeper-trains.
40. Anonymous, ‘Impact of COVID-19 on Global Tourism Made Clear as UNWTO Counts the Cost of Standstill’, *UNWTO*, 28 July 2020. https://www.unwto.org/news/impact-of-covid-19-on-global-tourism-made-clear-as-unwto-counts-the-cost-of-standstill; Stefan Gössling, Daniel Scott, and C. Michael Hall, ‘Pandemics, Tourism and Global Change: A Rapid Assessment of COVID-19’, *Journal of Sustainable Tourism*, Vol. 29, No. 1 (2020): 1–20. https://doi.org/10.1080/09669582.2020.1758708.
41. Dogan Gursoy and Christina Chi, ‘Effects of COVID-19 Pandemic on Hospitality Industry: Review of the Current Situations and a Research Agenda’, Vol. 29, No. 5 (2020): 527–529. https://doi.org/10.1080/19368623.2020.1788231.
42. Stefan Gössling, Daniel Scott, and C. Michael Hall, ‘Pandemics, Tourism and Global Change’, *Journal of Sustainable Tourism*, Vol. 29, No. 1: 1-20. https://doi.org/10.1080/09669582.2020.1758708.
43. Susan Houge Mackenzie and Jasmine Goodnow, ‘Adventure in the Age of COVID-19: Embracing Microadventures and Locavism in a Post-Pandemic World’, *Leisure Sciences*, published online on 3 July 2020. https://doi.org/10.1080/01490400.2020.1773984.
44. Kaitano Dube, Godwell Nhamo, and David Chikodzi, ‘COVID-19 Cripples Global Restaurant and Hospitality Industry’, *Current Issues in Tourism*, published online on 4 June 2020. https://doi.org/10.1080/13683500.2020.1773416.
45. A Marketing and Functions Coordinator at a restaurant in Melbourne, interview questions via personal correspondence, 21 October 2020.
46. Anonymous, ‘Chancellor Gives Diners 50% off on Eating Out’, *BBC News*, 8 July 2020. https://www.bbc.com/news/business-53337170.
47. Patrick Wood and Georgie Tunny, ‘From Brazil’s Farms to Melbourne’s Cafes, Coronavirus Is Crippling the Coffee Industry’, *ABC News*, 16 July 2020. https://www.abc.net.au/news/2020-07-17/brazil-farms-to-melbourne-cafes-coronavirus-cripples-coffee/12416166.
48. Nathaniel Meyersohn, ‘Coronavirus Will Change the Grocery Industry Forever’, *CNN*, 19 March 2020. https://www.cnn.com/2020/03/19/business/grocery-shopping-online-coronavirus/index.html; Leslie Wu, ‘Grocery Stores and the Effect of the COVID-19 Pandemic’, *Forbes*, 31 May 2020. https://www.forbes.com/sites/lesliewu/2020/05/31/grocery-stores-and-the-effect-of-the-covid-19-pandemic/; Anna Rahmanan, ‘How Covid-19 Has Changed Grocery Shopping’, *BBC*, 8 July 2020. https://www.bbc.com/worklife/article/20200707-how-covid-19-has-changed-grocery-shopping.
49. Kelly Cohen, ‘Tokyo 2020 Olympics Officially Postponed Until 2021’, *ESPN*, 25 March 2020. https://www.espn.com.au/olympics/story/_/id/28946033/tokyo-olympics-officially-postponed-2021.
50. Gabriel Baumgaertner, ‘Is Covid-19 Exposing MLB Players as Exploited Workers or Greedy Millionaires?’, *The Guardian*, 18 May 2020. http://www.theguardian.com/sport/2020/may/18/is-covid-19-exposing-mlb-players-as-exploited-workers-or-greedy-millionaires.
51. Anonymous, ‘Coronavirus Fears Abound as 66 Players Opt out of 2020 NFL Season’, *The Guardian*, 7 August 2020. http://www.theguardian.com/sport/2020/aug/06/nfl-players-opting-out-coronavirus-deadline.
52. Simon Grossobel, ‘How the Top European Football Leagues Are Dealing with Their 2019/20 Seasons’, *The National Law Review*, 14 May 2020. https://www.natlawreview.com/article/how-top-european-football-leagues-are-dealing-their-201920-seasons.
53. Beth G. Clarkson et al., ‘Covid-19: Reflections on Threat and Uncertainty for the Future of Elite Women’s Football in England’, *Managing Sport and Leisure*, published online on 14 May 2020. https://doi.org/10.1080/23750472.2020.1766377.
54. Jayashree Bhosale, ‘Prices of Agricultural Commodities Drop 20% Post COVID-19 Outbreak’, *The Economic Times*, 19 May 2020. https://economictimes.indiatimes.com/news/economy/agriculture/prices-of-agricultural-commodities-drop-20-post-covid-19-outbreak/articleshow/74705537.cms.
55. Alexandra Talty, ‘Volunteers Save New York’s Oldest Community Farm as Covid-19 Hits Agriculture’, *The Guardian*, 30 July 2020. http://www.theguardian.com/environment/2020/jul/30/csa-farms-covid-19-agriculture.
56. Darren Gray, ‘Coronavirus: Horticulture Giants Warn Fruit and Vegetable Prices Could Rise Due to Labour Shortage’, *The Sydney Morning Herald*, 30 July 2020. https://www.smh.com.au/business/companies/horticulture-giants-warn-fruit-and-vegetable-prices-could-rise-due-to-labour-shortage-20200729-p55gll.html.
57. Michael Hirtzer and Tatiana Freitas, ‘America Could Be Weeks from Meat Shortages with Shutdowns Spreading’, *Financial Post*, 24 April 2020. https://business.financialpost.com/commodities/agriculture/meat-supply-threats-grow-with-first-brazil-shutdown-u-s-turkey-halt; Ben Preiss and Benjamin Schneiders, ‘Meat Industry COVID-19 Cases Surge as Union Warns Against Shutdown’, *The Age*, 28 July 2020. https://www.theage.com.au/politics/victoria/meat-industry-covid-cases-surge-as-industry-contemplates-shutdown-20200728-p55g9g.html.
58. Maria Nicola et al., ‘The Socio-Economic Implications of the Coronavirus and COVID-19 Pandemic: A Review’, *International Journal of Surgery*, Vol. 78 (2020): 185–193. 10.1016/j.ijsu.2020.04.018.
59. Rick Brown and Amelia Hickman, ‘Changes in Online Gambling During the COVID-19 Pandemic’, *Australian Institute of Criminology*, 10 August 2020. https://www.aic.gov.au/publications/sb/sb25; Michael Auer, Doris Malischnig, and Mark D. Griffiths, ‘Gambling Before and During the COVID-19 Pandemic Among European Regular Sports Bettors: An Empirical Study Using Behavioral Tracking Data’, *International Journal of Mental Health and Addiction*, published online on 29 May 2020. 10.1007/s11469-020-00327-8; Anonymous, ‘Covid-19 Has Driven American Gamblers Online’, *The Economist*, 7 June 2020. https://www.economist.com/united-states/2020/06/07/covid-19-has-driven-american-gamblers-online.
60. Tahlea Aualiitia, ‘Pacific: Concerns Drug Traffickers Are Exploiting the Global Pandemic’, *ABC Radio Australia*, 4 August 2020. https://www.abc.net.au/radio-australia/programs/pacificbeat/pacific-drug-traffickers-exploiting-the-global-pandemic/12521094; Anonymous, ‘COVID-19 and the Drug Supply Chain: From Production and Trafficking to Use’, *Dovetail*, 15 May 2020. https://www.dovetail.org.au/news/2020/may/covid-19-and-the-drug-supply-chain-from-production-and-trafficking-to-use.
61. Emma Bubola, ‘“I Am Scared”: Italian Sex Workers Face Poverty and Illness in the Pandemic’, *The New York Times*, 3 August 2020. https://www.nytimes.com/2020/08/03/world/europe/italy-coronavirus-prostitution-sex-work.html; Kate Connolly, ‘Germany’s Sex Workers Demand Easing of Covid-19 Restrictions’, *The Guardian*, 6 August 2020. https://www.theguardian.com/world/2020/aug/06/germany-sex-workers-demand-easing-of-covid-19-restrictions; Roxana Diamond, ‘How the “National Cabinet of Whores” Is Leading Australia’s Coronavirus Response for Sex Workers’, *The Conversation*, 7 August 2020. http://theconversation.com/how-the-national-cabinet-of-whores-is-leading-australias-coronavirus-response-for-sex-workers-142833; Jacqueline Lewis, ‘Sex Workers Are Criminalized and Left Without Government Support During the Coronavirus Pandemic’, *The Conversation*, 21 July 2020. http://theconversation.com/sex-workers-are-criminalized-and-left-without-government-support-during-the-coronavirus-pandemic-141746.
62. Anonymous, ‘COVID-19 Impact and Response for Sex Workers’, *Scarlet Alliance*, 2020. http://www.scarletalliance.org.au/COVID-19/.
63. Helena Fitzgerald, ‘Coronavirus Risk Doesn’t Stop at Your Front Door’, *The Atlantic*, 15 March 2020. https://www.theatlantic.com/family/archive/2020/03/what-does-social-distance-mean-within-a-family/608044/.
64. A grandmother in California, video interview, 17 October 2020.
65. A grandmother in Italy, interview questions via personal correspondence, 22 October 2020 (Translated from Italian into English by the authors).
66. Katherine Ellison, ‘Perspective | Stress from the Pandemic Can Destroy Relationships with Friends—Even Families’, *The Washington Post*, 8 August 2020. https://www.washingtonpost.com/health/stress-from-the-pandemic-can-destroy-relationships-with-friends--even-families/2020/08/07/d95216f4-d665-11ea-aff6-220dd3a14741_story.html.
67. Samantha Selinger-Morris, ‘Has the Pandemic Given New Meaning to Your Friendships?’, *The Sydney Morning Herald*, 17 May 2020. https://www.smh.com.au/lifestyle/life-and-relationships/has-the-pandemic-given-new-meaning-to-your-friendships-20200515-p54tae.html.
68. Anonymous, ‘Staying Connected While Being Physically Apart: Wellbeing in the Time of Social Distancing’, *The Government of Queensland*, 27 March 2020. https://www.health.qld.gov.au/news-events/news/staying-connected-while-social-distancing-isolation-loneliness-technology-physical-connection-community-family-friends.
69. Sophie Aubrey, ‘Coronavirus Australia: Older Australians Most Isolated, But YouTube Grandmother Offers Hope’, *The Sydney Morning Herald*, 30 July 2020. https://www.smh.com.au/lifestyle/life-and-relationships/older-australians-most-isolated-during-covid-19-but-youtube-nanna-offers-hope-20200729-p55gmc.html.
70. Olivia Willis and Paige Cockburn, ‘The Ways Young People Are Adapting to Pandemic Life’, *ABC News*, 11 July 2020. https://www.abc.net.au/news/health/2020-07-12/how-young-people-cope-and-adapt-during-coronavirus-pandemic/12437896.
71. S. Harris Ali and Fuyuki Kurasawa, ‘#COVID19: Social Media Both a Blessing and a Curse During Coronavirus Pandemic’, *The Conversation*, 22 March 2020. http://theconversation.com/covid19-social-media-both-a-blessing-and-a-curse-during-coronavirus-pandemic-133596.
72. Jessie Davies, ‘Dating Apps Notice Traffic Bump Amid Coronavirus Social-Distancing’, *ABC News*, 2 April 2020. https://www.abc.net.au/news/2020-04-03/online-dating-in-the-age-of-coronavirus/12102282.
73. Susan S. Hendrick and Clyde Hendrick, ‘Romantic Love in the Age of COVID-19’, *Journal of Loss and Trauma*, Vol. 25, No. 6 (2020): 540–543. https://doi.org/10.1080/15325024.2020.1763557.
74. Kristina Coop Gordon and Erica A. Mitchell, ‘Infidelity in the Time of COVID-19’, *Family Process*, Vol. 59, No. 3 (2020): 956–966. 10.1111/famp.12576.
75. Yara Murray-Atfield and Joseph Dunstan, ‘Melbourne Placed Under Stage 4 Coronavirus Lockdown, Stage 3 for Rest of Victoria as State of Disaster Declared’, *ABC News*, 2 August 2020. https://www.abc.net.au/news/2020-08-02/victoria-coronavirus-restrictions-imposed-death-toll-cases-rise/12515914.
76. Samantha Selinger-Morris, ‘Coronavirus Australia: Pet Adoption on the Rise During COVID-10 Pandemic’, *The Sydney Morning Herald*, 26 April 2020. https://www.smh.com.au/lifestyle/life-and-relationships/they-ve-wanted-a-dog-for-years-lockdown-has-finally-made-it-happen-20200424-p54n0n.html; L. F. Carver, ‘How the Coronavirus Pet Adoption Boom Is Reducing Stress’, *The Conversation*, 24 May 2020. http://theconversation.com/how-the-coronavirus-pet-adoption-boom-is-reducing-stress-138074.
77. Rachael Conaghan, ‘People Angry That Racing Can Continue During Victorian Restrictions’, *Junkee*, 4 August 2020. https://junkee.com/victoria-stage-four-racing/264633.
78. Katy Ramsey Mason, ‘Landlord-Leaning Eviction Courts Are About to Make the Coronavirus Housing Crisis a Lot Worse’, *The Conversation*, 29 July 2020. http://theconversation.com/landlord-leaning-eviction-courts-are-about-to-make-the-coronavirus-housing-crisis-a-lot-worse-142803.
79. Ganesh Setty, ‘Tenant Decapitated Landlord with a Sword over a Rent Dispute, Hartford Police Say’, *CNN*, 31 July 2020. https://edition.cnn.com/2020/07/31/us/hartford-sword-homicide-trnd/index.html.
80. Caroline Bradbury-Jones and Louise Isham, ‘The Pandemic Paradox: The Consequences of COVID-19 on Domestic Violence’, *Journal of Clinical Nursing*, Vol. 29, No. 14 (2020): 2047–2049. 10.1111/jocn.15296.
81. Alex R. Piquero et al., ‘Staying Home, Staying Safe? A Short-Term Analysis of COVID-19 on Dallas Domestic Violence’, *American Journal of Criminal Justice*, Vol. 45 (2020): 601–635. 10.1007/s12103-020-09531-7.
82. James M. Clay and Matthew O. Parker, ‘Alcohol Use and Misuse During the COVID-19 Pandemic: A Potential Public Health Crisis?’, *The Lancet Public Health*, Vol. 5, No. 5 (2020): e259. 10.1016/S2468-2667(20)30088-8.
83. Patrick Hatch, ‘Illegal Online Casinos Boom During COVID-19 Lockdown’, *The Sydney Morning Herald*, 17 June 2020. https://www.smh.com.au/business/companies/illegal-online-casinos-boom-during-covid-19-lockdown-20200616-p552yl.html.
84. Matteo Bonotti et al., ‘COVID-19 in Everyday Spaces: Social and Political Considerations’, *ABC Religion & Ethics*, 17 June 2020. https://www.abc.net.au/religion/covid19-in-everyday-spaces-and-social-division/12365294.
85. Russell M. Viner et al., ‘School Closure and Management Practices During Coronavirus Outbreaks Including COVID-19: A Rapid Systematic Review’, *The Lancet Child & Adolescent Health*, Vol. 4, No. 5 (2020): 397–404. 10.1016/S2352-4642(20)30095-X.
86. Jon Pedersen, ‘Will COVID-19 Be the Death of Summer Vacation?’, *The Conversation*, 13 April 2020. http://theconversation.com/will-covid-19-be-the-death-of-summer-vacation-135776.
87. A teacher in Italy, interview questions via personal correspondence, 3 November 2020 (Translated from Italian into English by the authors).
88. Anonymous, ‘COVID-19 on Campus: How Should Schools Be Redesigned?’, *Wharton University of Pennsylvania*, 22 June 2020. https://knowledge.wharton.upenn.edu/article/how-should-universities-be-redesigned-in-the-wake-of-covid-19/.
89. Scott Galloway, ‘Colleges Must Think Like Businesses to Survive COVID-19’, *Business Insider*, 18 July 2020. https://www.businessinsider.com/scott-galloway-colleges-must-cut-costs-to-survive-covid-2020-7?r=AU&IR=T.
90. Leslie Wu, ‘What Covid-19 Social Distancing Measures Will Mean For Restaurant Dining Room Design’, *Forbes*, 27 May 2020. https://www.forbes.com/sites/lesliewu/2020/05/27/what-covid-19-social-distancing-measures-will-mean-for-restaurant-dining-room-design/#14d612f52200; James Ellerby, ‘Seven Ways Social Distancing Will Change Restaurants’, *The Conversation*, 1 July 2020. http://theconversation.com/seven-ways-social-distancing-will-change-restaurants-141671.
91. Chris McGonigal, ‘10 Photos Show How Restaurants Around The World Are Social Distancing’, *Huffington Post*, 13 May 2020. https://www.huffpost.com/entry/photos-restaurants-social-distancing-coronavirus_l_5ebc0589c5b6ac90c5bafb67.
92. Lora Jones, ‘Coronavirus: When and How Are Pubs Allowed to Open?’, *BBC News*, 14 October 2020. https://www.bbc.com/news/business-52977388.
93. Jessica Hinchliffe, ‘Did Coronavirus Kill the Humble Beer Coaster?’, *ABC News*, 18 July 2020. https://www.abc.net.au/news/2020-07-19/covid19-spells-the-end-of-the-beer-coaster-pubc/12467108.
94. Summer Woolley, ‘Pub “bans” Neil Diamond’s Hit Song Due to COVID Risk’, *7NEWS*, 4 August 2020. https://7news.com.au/lifestyle/health-wellbeing/pub-bans-neil-diamonds-sweet-caroline-during-covid-pandemic-c-1214745.
95. Matt Bungard and Laura Chung, ‘Coronavirus Australia: Sydney Cafes Innovate Amid COVID-19 Lockdown Restrictions’, *The Sydney Morning Herald*, 6 May 2020. https://www.smh.com.au/business/consumer-affairs/from-coffees-to-groceries-sydney-cafes-innovate-amid-covid-19-lockdown-restrictions-20200424-p54n0t.html.
96. Connor McCallum, ‘COVID Has Wounded America’s Coffee Culture, and We Could Be Next’, *The Big Smoke*, 4 July 2020. https://www.thebigsmoke.com.au/2020/07/04/covid-has-wounded-americas-coffee-culture-and-we-could-be-next/.
97. Anonymous, ‘The Community Roles of the Barber Shop and Beauty Salon’, *National Museum of African American History and Culture*, n.d. https://nmaahc.si.edu/blog/community-roles-barber-shop-and-beauty-salon; Patricia Burke Wood and Rod K. Brunson, ‘Geographies of Resilient Social Networks: The Role of African American Barbershops’, *Urban Geography*, Vol. 32, No. 2 (2013): 228–243. https://doi.org/10.2747/0272-3638.31.2.228.
98. Kevin Armstrong, ‘A South Bronx Barber Takes on a New Role in His Pandemic-Ravaged Community’, *The Washington Post*, 18 July 2020. https://www.washingtonpost.com/national/a-south-bronx-barber-takes-on-a-new-role-in-his-pandemic-ravaged-community/2020/07/17/dcd645ac-c2b0-11ea-b178-bb7b05b94af1_story.html.
99. Nkayla Afshariyan, ‘Barbers Want Their Shops to Be Safe Spaces for Men’s Mental Health’, *Triple J*, 24 April 2020. https://www.abc.net.au/triplej/programs/hack/might-and-mane-mental-health-training-for-barbers/11022744.
100. Lauren Valenti, ‘How Hair Salons Will Be Transformed by the Global Pandemic’, *Vogue*, 2 May 2020. https://www.vogue.com/article/hair-salons-covid-19-global-pandemic.
101. Hannah McCann, ‘Coronavirus Shutdowns: What Makes Hairdressing “Essential”? Even the Hairdressers Want to Close’, *The Conversation*, 15 April 2020. https://theconversation.com/coronavirus-shutdowns-what-makes-hairdressing-essential-even-the-hairdressers-want-to-close-135811.
102. Brett Worthington, ‘“Just Bonkers”: Hairdressers Slam Reversal of 30-Minute Time Limit and Lack of Shutdown’, *ABC News*, 25 March 2020. https://www.abc.net.au/news/2020-03-26/coronavirus-hairdresser-limit-to-be-scrapped/12091700.
103. Moriah Balingit, ‘Armed Militia Helped a Michigan Barbershop Open, a Coronavirus Defiance That Puts Republican Lawmakers in a Bind’, *The Washington Post*, 13 May 2020. https://www.washingtonpost.com/national/coronavirus-michigan-republicans-whitmer/2020/05/12/54975e1a-9466-11ea-82b4-c8db161ff6e5_story.html.
104. Australian Government Department of Health, ‘Exercising and Staying Active During Coronavirus (COVID-19) Restrictions’, *Australian Government Department of Health*, 11 May 2020. https://www.health.gov.au/news/health-alerts/novel-coronavirus-2019-ncov-health-alert/ongoing-support-during-coronavirus-covid-19/exercising-and-staying-active-during-coronavirus-covid-19-restrictions; Anonymous, ‘#HealthyAtHome—Physical Activity’, *World Health Organisation*, 2020. https://www.who.int/news-room/campaigns/connecting-the-world-to-combat-coronavirus/healthyathome/healthyathome---physical-activity.
105. Francesca Gillett, ‘Coronavirus: How Joe Wicks’ Fitness Workouts “Changed My Life”’, *BBC News*, 22 July 2020. https://www.bbc.com/news/uk-53487312.
106. Adriane Reardon and Timothy Fernandez, ‘“It’s Been Very Full on”: 24/7 Gyms Feel the Pinch as New COVID-19 Restrictions Take Effect’, *ABC News*, 1 August 2020. https://www.abc.net.au/news/2020-08-01/nsw-gyms-require-coronavirus-marshal/12514014; Mimi Becker, ‘Timeline for Gyms Reopening Across Australia’, *9Now*, 20 May 2020. https://9now.nine.com.au/a-current-affair/coronavirus-gyms-reopening-across-australia-in-may-and-june-how-they-plan-to-minimise-covid19-risk/66a89335-d5f8-4f6f-bfc8-0ce2b520b2be; Jacob McMaster and Ferguson Claudia, ‘COVID-19 Rules Tighten for Gyms’, *Narooma News*, 7 August 2020. https://www.naroomanewsonline.com.au/story/6866646/covid-19-rules-tighten-for-gyms/.
107. Anonymous, ‘Massive Boost to Outdoor Exercise in Sydney’s Green Spaces During COVID-19, Survey Finds’, *ABC News*, 28 June 2020. https://www.abc.net.au/news/2020-06-28/sydneys-green-space-use-booms-during-covid-19/12400104.
108. Timothy Walsh and Deborah Glasofer, ‘Raising a Teenager with an Eating Disorder in a Pandemic’, *OUPblog*, 7 August 2020. https://blog.oup.com/2020/08/raising-a-teenager-with-an-eating-disorder-in-a-pandemic/; Achraf Ammar et al., ‘Effects of COVID-19 Home Confinement on Eating Behaviour and Physical Activity: Results of the ECLB-COVID19 International Online Survey’, *Nutrients*, Vol. 12, No. 6 (2020): 1583. 10.3390/nu12061583; Andrea Phillipou et al., ‘Eating and Exercise Behaviors in Eating Disorders and the General Population During the COVID-19 Pandemic in Australia: Initial Results from the COLLATE Project’, *International Journal of Eating Disorders*, Vol. 53, No. 7 (2020): 1158–1165. 10.1002/eat.23317.
109. Liffy Thomas, ‘How Meetup Groups Are Staying in Touch Through Virtual Platforms’, *The Hindu*, 3 August 2020. https://www.thehindu.com/news/cities/chennai/how-meetup-groups-are-staying-in-touch-through-virtual-platforms/article32258295.ece.
110. Lourdes Zamanillo Tamborrel, interview questions via personal correspondence, 2 November 2020. For additional details see Lourdes Zamanillo Tamborrel, ‘Meetup.com: A Cohesive Tool for Multicultural Societies?’, PhD thesis in progress, Monash University, Melbourne.
111. Kenneth Kiesnoski, ‘Travel Changed After 9/11; Here’s How It Will Look After the Covid-19 Pandemic Finally Recedes’, *CNBC*, 10 May 2020. https://www.cnbc.com/2020/05/10/heres-how-travel-will-change-after-the-covid-19-pandemic-recedes.html.
112. Ben Smee, ‘Bondi Beach Closed After Crowds Defy Ban on Gatherings of 500-Plus’, *The Guardian*, 21 March 2020. https://www.theguardian.com/australia-news/2020/mar/21/bondi-beach-closed-down-after-crowds-defy-ban-on-gatherings-of-more-than-500-people; Angela Giuffrida, ‘Coronavirus Brings Tension and Prejudice to Italy’s Beaches’, *The Guardian*, 12 July 2020. https://www.theguardian.com/world/2020/jul/12/coronavirus-brings-tension-prejudice-italy-beaches; Anonymous, ‘War, Algae, Virus: The Beach That Won’t Be Beaten’, *BBC News*, 15 June 2020. https://www.bbc.com/news/av/world-europe-53054161.
113. Peter Hannam, ‘Visits to NSW National Parks Soar During COVID-19’, *The Sydney Morning Herald*, 5 August 2020. https://www.smh.com.au/environment/conservation/good-for-the-soul-visits-to-nsw-national-parks-soar-amid-covid-19-20200805-p55iu8.html; Lauren Roberts and Henry Zwartz, ‘Uluru-Kata Tjuta National Park Closure Extended Out of “Utmost Respect” Amid Coronavirus Concerns’, *ABC News*, 4 August 2020. https://www.abc.net.au/news/2020-08-04/coronavirus-fears-force-closure-of-uluru/12521324.
114. Vikas Mehta, ‘The New Proxemics: COVID-19, Social Distancing, and Sociable Space’, *Journal of Urban Design*, published online on 2 July 2020. https://doi.org/10.1080/13574809.2020.1785283.
115. Setha Low and Alan Smart, ‘Thoughts About Public Space During Covid-19 Pandemic’, *City & Society*, Vol. 32, No. 1 (2020). 10.1111/ciso.12260.
116. Selby Coxon et al., ‘Rethinking Public Transport Post-Pandemic’, *Monash Lens*, 3 June 2020. https://lens.monash.edu/@politics-society/2020/06/03/1380582?slug=returning-confidence-in-public-transport-in-a-post-covid-19-pandemic-world.
117. Peter Sweatman and Majid Sarvi, ‘Covid-19: A Challenge and an Opportunity for Public Transport’, *Pursuit*, 3 August 2020. https://pursuit.unimelb.edu.au/articles/covid-19-a-challenge-and-an-opportunity-for-public-transport.
118. Aimee Chanthadavong, ‘Victorian Government Trials Touchless Pedestrian Crossing Technology in Melbourne’, *ZDNet*, 30 July 2020. https://www.zdnet.com/article/victorian-government-trials-touchless-pedestrian-crossing-technology-in-melbourne/.
119. Roland Geraerts, ‘Coronavirus: Using Crowd Simulation to Encourage Social Distancing’, *The Conversation*, 4 August 2020. http://theconversation.com/coronavirus-using-crowd-simulation-to-encourage-social-distancing-143121.
120. Eliza W. Kinsey, Dirk Kinsey, and Andrew G. Rundle, ‘COVID-19 and Food Insecurity: An Uneven Patchwork of Responses’, *Journal of Urban Health: Bulletin of the New York Academy of Medicine*, Vol. 97, No. 3 (2020): 332–335. 10.1007/s11524-020-00455-5.
121. Coleen A. Boyle et al., ‘The Public Health Response to the COVID-19 Pandemic for People with Disabilities’, *Disability and Health Journal*, Vol. 13, No. 3 (2020): 100943. 10.1016/j.dhjo.2020.100943.
122. Nguyen Ngoc Long and Bui Huy Khoi, ‘An Empirical Study About the Intention to Hoard Food During COVID-19 Pandemic’, *EURASIA Journal of Mathematics*, *Science and Technology Education*, Vol. 16, No. 7 (2020): em1857. 10.29333/ejmste/8207; Benjamin Oosterhoff and Cara Palmer, ‘Psychological Correlates of News Monitoring, Social Distancing, Disinfecting, and Hoarding Behaviors Among US Adolescents During the COVID-19 Pandemic’, *PsyArXiv*, published online on 23 March 2020. 10.31234/osf.io/rpcy4; Stephanie Preston, ‘Your Brain Evolved to Hoard Supplies and Shame Others for Doing the Same’, *The Conversation*, 27 March 2020. http://theconversation.com/your-brain-evolved-to-hoard-supplies-and-shame-others-for-doing-the-same-134634; Conor Wynn, ‘Understanding Personality May Explain Why Some of Us Are More Prone to Toilet Paper Panic’, *ABC News*, 24 March 2020. https://www.abc.net.au/news/2020-03-25/coronavirus-personality-types-toilet-paper/12085776; Eric Dolan, ‘Narcissistic Personalities Linked to Defiance of Coronavirus Prevention Guidelines and Hoarding’, *PsyPost*, 21 July 2020. https://www.psypost.org/2020/07/narcissistic-personalities-linked-to-defiance-of-coronavirus-prevention-guidelines-and-hoarding-57230.
123. Kate Berg, ‘Lansing Man Dies After Being Stabbed at Quality Dairy in Mask Dispute’, *Lansing State Journal*, 10 August 2020. https://www.lansingstatejournal.com/story/news/2020/08/10/lansing-man-dies-after-being-stabbed-quality-dairy-mask-dispute/3333509001/.
124. Brad A. Meisner, ‘Are You OK, Boomer? Intensification of Ageism and Intergenerational Tensions on Social Media Amid COVID-19’, *Leisure Sciences*, published online on 24 June 2020. https://doi.org/10.1080/01490400.2020.1773983.
125. Soutik Biswass, ‘India Coronavirus: “Our Neighbours Made Us Covid-19 Pariahs”’, *BBC News*, 19 July 2020. https://www.bbc.com/news/world-asia-india-53418762.
126. Chin Tan, ‘Of All the Coronavirus Racist Attacks We’ve Seen, One Story Struck Me the Most’, *ABC News*, 9 May 2020. https://www.abc.net.au/news/2020-05-09/coronavirus-covid-19-racist-attacks-data-collection-strategy/12229162.
127. Anonymous, ‘Covid-19 Fueling Anti-Asian Racism and Xenophobia Worldwide’, *Human Rights Watch*, 12 May 2020. https://www.hrw.org/news/2020/05/12/covid-19-fueling-anti-asian-racism-and-xenophobia-worldwide; Asian Australian Alliance and Osmond Chiu, ‘COVID-19 Coronavirus Racism Incident Report’, *Australian Asian Alliance*, 24 April 2020. http://diversityarts.org.au/app/uploads/COVID19-racism-incident-report-Preliminary-Official.pdf.
128. Imran Awan, ‘Coronavirus: Conspiracy Theories and Fake Videos Fuel Rise in Islamophobia’, *The Conversation*, 25 April 2020. http://theconversation.com/coronavirus-conspiracy-theories-and-fake-videos-fuel-rise-in-islamophobia-137107; Anonymous, ‘Coronavirus, Fear and How Islamophobia Spreads on Social Media’, *Anti*-*Muslim Hatred Working Group*, 20 April 2020. https://anti-muslim-hatred-working-group.home.blog/2020/04/20/coronavirus-fear-and-how-islamophobia-spreads-on-social-media/.
129. Stuart Winer, ‘COVID-19 Fueling Worldwide Wave of Anti-Semitism, Researchers Find’, *The Times of Israel*, 23 June 2020. https://www.timesofisrael.com/covid-19-fueling-worldwide-wave-of-anti-semitism-researchers-find/.
130. Margareta Matache and Jacqueline Bhabha, ‘Anti-Roma Racism Is Spiraling During COVID-19 Pandemic’, *Health and Human Rights*, Vol. 22, No. 1 (2020): 379–382. https://www.ncbi.nlm.nih.gov/pmc/articles/PMC7348427/.
131. Piotr Sorokowski et al., ‘Can Information About Pandemics Increase Negative Attitudes Toward Foreign Groups? A Case of COVID-19 Outbreak’, *Sustainability*, Vol. 12, No. 12 (2020): 4912. 10.3390/su12124912.
132. Bumsoo Kim, ‘Effects of Social Grooming on Incivility in COVID-19’, *Cyberpsychology, Behavior, and Social Networking*, Vol. 23, No. 8 (2020): 19. 10.1089/cyber.2020.0201.
133. François Heisbourg, ‘From Wuhan to the World: How the Pandemic Will Reshape Geopolitics’, *Survival*, Vol. 62, No. 3 (2020): 7–24. https://doi.org/10.1080/00396338.2020.1763608.
134. Anonymous, ‘COVID-19 and Conflict: Seven Trends to Watch’, *Crisis Group*, 24 March 2020. https://www.crisisgroup.org/global/sb4-covid-19-and-conflict-seven-trends-watch; Colum Lynch and Robbie Gramer, ‘U.S. and China Turn Coronavirus Into a Geopolitical Football’, *Foreign Policy*, 11 March 2020. https://foreignpolicy.com/2020/03/11/coronavirus-geopolitics-china-united-states-trump-administration-competing-global-health-response/.
135. Anonymous, ‘Coronavirus: US “Wants 3M to End Mask Exports to Canada and Latin America”’, *BBC News*, 2 April 2020. https://www.bbc.com/news/world-us-canada-52161032.
136. Thomas J. Bollyky and Chad P. Bown, ‘The Tragedy of Vaccine Nationalism’, *Foreign Affairs*, 6 October 2020. https://www.foreignaffairs.com/articles/united-states/2020-07-27/vaccine-nationalism-pandemic.
137. Paul Staniland, ‘Kashmir, India, and Pakistan and Coronavirus—Coronavirus in Conflict Zones: A Sobering Landscape’, *Carnegie Endowment for International Peace*, 14 April 2020. https://carnegieendowment.org/2020/04/14/kashmir-india-and-pakistan-and-coronavirus-pub-81529.
138. Gavin Lee, ‘Coronavirus: EU Leaders Reach Recovery Deal After Marathon Summit’, *BBC News*, 21 July 2020. https://www.bbc.com/news/world-europe-53481542.
139. Rory Carroll Sam Jones in Madrid et al., ‘Covid-19 Crisis Stokes European Tensions over Migrant Labour’, *The Guardian*, 11 May 2020. https://www.theguardian.com/world/2020/may/11/covid-19-crisis-stokes-european-tensions-over-migrant-labour.
140. Anonymous, ‘Migrants and Refugees Are Being Forgotten in the COVID-19 Response. This Has to Change’, *Europeansting*, 13 August 2020. https://europeansting.com/2020/08/13/migrants-and-refugees-are-being-forgotten-in-the-covid-19-response-this-has-to-change/; Nassim Khadem, ‘Coronavirus Recession Puts Thousands of Refugees and Asylum Seekers at Risk of Job Loss, Homelessness’, *ABC News*, 30 July 2020. https://www.abc.net.au/news/2020-07-30/coronavirus-recession-refugees-asylum-seekers-at-risk-homeless/12503874.
141. *The Lancet*, ‘Humanitarian Crises in a Global Pandemic’, *The Lancet*, Vol. 396, No. 10249 (2020): 447. 10.1016/S0140-6736(20)31749-9.
142. Tom Staynor, ‘Treasury Figures Show Temporary Visa Holders Are Being Disproportionately Hurt by Coronavirus Lay-Offs’, *SBS News*, 23 July 2020. https://www.sbs.com.au/news/treasury-figures-show-temporary-visa-holders-are-being-disproportionately-hurt-by-coronavirus-lay-offs.
143. Anonymous, ‘Trump Considers Banning Re-Entry by Citizens Who May Have Coronavirus’, *The Indian Express*, 11 August 2020. https://indianexpress.com/article/coronavirus/trump-considers-banning-re-entry-by-citizens-who-may-have-coronavirus-6549731/.
144. Anonymous, ‘USCIS Response to COVID-19’, *U.S. Citizenship and Immigration Services*, 7 October 2020. https://www.uscis.gov/about-us/uscis-response-to-covid-19.
145. Anonymous, ‘Coronavirus Government Response Tracker’, *Blavatnik School of Government*, 14 September 2020. https://www.bsg.ox.ac.uk/research/research-projects/coronavirus-government-response-tracker.
146. Mark Leonard, ‘The Brexit Parenthesis: Three Ways the Pandemic Is Changing UK Politics’, *European Council of Foreign Relations*, 12 August 2020. https://www.ecfr.eu/publications/summary/the_brexit_parenthesis_three_ways_the_pandemic_is_changing_uk_politics.
147. Toluse Olorunnipa, ‘Trump Cites Game Show Host on Pandemic While Undercutting Doctors and Questioning Their Expertise’, *The Washington Post*, 14 July 2020. https://www.washingtonpost.com/politics/trump-cites-game-show-host-on-pandemic-while-undercutting-doctors-and-questioning-their-expertise/2020/07/13/a083ea5c-c51f-11ea-8ffe-372be8d82298_story.html.
148. Lauren Aratani, ‘How Did Face Masks Become a Political Issue in America?’, *The Guardian*, 29 June 2020. http://www.theguardian.com/world/2020/jun/29/face-masks-us-politics-coronavirus.
149. Julie Jiang et al., ‘Political Polarization Drives Online Conversations About COVID-19 in the United States’, *Human Behavior and Emerging Technologies*, Vol. 2, No. 3 (2020): 200–211. 10.1002/hbe2.202; Ashley Quarcoo and Rachel Kleinfeld, ‘Can the Coronavirus Heal Polarization?’, *Carnegie Endowment for International Peace*, 1 May 2020. https://carnegieendowment.org/2020/05/01/can-coronavirus-heal-polarization-pub-81704.
150. Megan Janetsky and Anthony Faiola, ‘Colombian Guerrillas Are Using Coronavirus Curfews to Expand Their Control. Violators Have Been Killed’, *The Washington Post*, 26 July 2020. https://www.washingtonpost.com/world/the_americas/colombia-coronavirus-farc-eln-guerrillas/2020/07/25/927d3c06-cb64-11ea-bc6a-6841b28d9093_story.html.
151. Caleb Weiss, ‘Jihadists Discuss Coronavirus, Offer Advice’, *FDD’s Long War Journal*, published online on 13 March 2020. https://www.longwarjournal.org/archives/2020/03/jihadists-discuss-coronavirus-offer-advice.php; Gregoire Phillips, ‘As Governments Dither on COVID-19, Jihadists and Gang Leaders Step In’, *Political Violence at a Glance*, 15 April 2020. https://politicalviolenceataglance.org/2020/04/15/as-governments-dither-on-covid-19-jihadists-and-gang-leaders-step-in/.
152. Kate Connolly, ‘Berlin Protests Against Coronavirus Rules Divide German Leaders’, *The Guardian*, 3 August 2020. http://www.theguardian.com/world/2020/aug/03/berlin-protests-against-coronavirus-rules-divide-german-leaders.
153. Anonymous, ‘CoronaCheck: Is Victoria’s COVID-19 Increase Linked to the Black Lives Matter Protests?’, *ABC News*, 25 June 2020. https://www.abc.net.au/news/2020-06-26/coronacheck-victoria-black-lives-matter-protests-family-spike/12391628.
154. Laignee Barron, ‘Lessons From Hong Kong’s Decision to Postpone Elections’, *Time*, 7 August 2020. https://time.com/5877242/coronavirus-elections-postpone-delay-hong-kong-covid19/.
155. Kate Rabinowitz and Brittany Renee Mayes, ‘At Least 84% of American Voters Can Cast Ballots By Mail In the Fall’, *The Washington Post*, 25 September 2020. https://www.washingtonpost.com/graphics/2020/politics/vote-by-mail-states/; Anonymous, ‘Postal Voting for Local Government Elections on October 24’, *The Wimmera Mail*-*Times*, 15 May 2020. https://www.mailtimes.com.au/story/6758651/postal-voting-for-local-government-elections-on-october-24/.
156. Anonymous, ‘Singapore to Hold General Elections Amid Covid-19: What Is at Stake?’, *The Indian Express*, 25 June 2020. https://indianexpress.com/article/explained/singapore-general-elections-coronavirus-6473772/; Christopher Thomas, ‘Elections and COVID-19: Ensuring Safety in the Voting Process’, *Esri*, 21 April 2020. https://www.esri.com/about/newsroom/blog/covid-19-social-distancing-elections-process/.
157. Yvette Tan, ‘Coronavirus in Singapore: Election Campaigning Without the Handshakes’, *BBC News*, 5 July 2020. https://www.bbc.com/news/world-asia-53216390.
158. Sean Murphy, ‘Health Official: Trump Rally “Likely” Source of Virus Surge’, *AP NEWS*, 8 July 2020, https://apnews.com/article/ad96548245e186382225818d8dc416eb; Anonymous, ‘Trump Rally a “Dangerous Move” as Coronavirus Cases Spike in US’, *The Sydney Morning Herald*, 14 June 2020. https://www.smh.com.au/world/north-america/trump-rally-a-dangerous-move-as-coronavirus-cases-spike-in-us-20200615-p552ke.html.
159. Anonymous, ‘White House Rally: Trump Holds First Public Event Since Covid Diagnosis’, *BBC News*, 10 October 2020. https://www.bbc.com/news/election-us-2020-54493575.
160. Barbara Sprunt, ‘Harris, as Biden’s Running Mate, Says Case Against Trump Is “Open And Shut”’, *NPR*, 12 August 2020. https://www.npr.org/2020/08/12/901462712/biden-and-harris-to-introduce-their-presidential-ticket-in-delaware.
161. Molly Ball, ‘Donald Trump’s COVID-19 Diagnosis Is Forcing Him to Face His Personal—and Political—Vulnerability’, *Time*, 8 October 2020. https://time.com/5897886/trump-coronavirus/.
162. Peter McCutcheon, ‘How COVID-19 Is Changing the Political Debate on Queensland’s Debt’, *ABC News*, 25 July 2020. https://www.abc.net.au/news/2020-07-25/coronavirus-queensland-debt-analysis-election-alp-lnp/12488184.
163. Daisy Fancourt, Andrew Steptoe, and Liam Wright, ‘The Cummings Effect: Politics, Trust, and Behaviours During the COVID-19 Pandemic’, *The Lancet*, Vol. 396, No. 10249 (2020): 464–465. 10.1016/S0140-6736(20)31690-1.
164. Toluse Olorunnipa, ‘Trump Cites Game Show Host on Pandemic While Undercutting Doctors and Questioning Their Expertise’, *The Washington Post*, 14 July 2020. https://www.washingtonpost.com/politics/trump-cites-game-show-host-on-pandemic-while-undercutting-doctors-and-questioning-their-expertise/2020/07/13/a083ea5c-c51f-11ea-8ffe-372be8d82298_story.html.
165. Simone Fox Koob and Jewel Topsfield, ‘Mask-Dodging Woman Allegedly Smashed Female Cop’s Head into Concrete’, *The Age*, 4 August 2020. https://www.theage.com.au/national/victoria/mask-dodging-woman-allegedly-smashed-female-cop-s-head-into-concrete-20200804-p55ica.html.
166. Hannah Allam, ‘Michigan Domestic Terror Plot Sends Shockwaves Through Militia World’, *NPR*, 9 October 2020. https://www.npr.org/2020/10/09/922319136/michigan-domestic-terror-plot-sends-shockwaves-through-militia-world.
167. Denis Muller, ‘Tensions Rise on Coronavirus Handling as the Media Take Control of the Accountability Narrative’, *The Conversation*, 11 August 2020 http://theconversation.com/tensions-rise-on-coronavirus-handling-as-the-media-take-control-of-the-accountability-narrative-144195.
168. Mark Hertsgaard, ‘The Media’s Covid-19 Coverage Proves It Could Also Spotlight the Climate Crisis’, *The Nation*, 25 March 2020. https://www.thenation.com/article/environment/coronavirus-media-climate/.
169. Heather Kelly, ‘Facebook Removes a Coronavirus Misinformation Post from Trump for the First Time Ever’, *The Washington Post*, 8 August 2020. https://www.washingtonpost.com/technology/2020/08/05/trump-post-removed-facebook/.

